# Enhanced multimodal biometric recognition approach for smart cities based on an optimized fuzzy genetic algorithm

**DOI:** 10.1038/s41598-021-04652-3

**Published:** 2022-01-12

**Authors:** Vani Rajasekar, Bratislav Predić, Muzafer Saracevic, Mohamed Elhoseny, Darjan Karabasevic, Dragisa Stanujkic, Premalatha Jayapaul

**Affiliations:** 1 Department of CSE, Kongu Engineering College, Perundurai, Erode, India; 2 Faculty of Electronic Engineering, University of Niš, Niš, Serbia; 3 Department of Computer Sciences, University of Novi Pazar, Novi Pazar, Serbia; 4 College of Computing and Informatics, University of Sharjah, Dubai, UAE; 5 Faculty of Applied Management, Economics and Finance, University Business Academy in Novi Sad, Belgrade, Serbia; 6 Technical Faculty in Bor, University of Belgrade, Belgrade, Serbia; 7 Department of IT, Kongu Engineering College, Perundurai, Erode, India

**Keywords:** Computer science, Information technology, Software

## Abstract

Biometric security is a major emerging concern in the field of data security. In recent years, research initiatives in the field of biometrics have grown at an exponential rate. The multimodal biometric technique with enhanced accuracy and recognition rate for smart cities is still a challenging issue. This paper proposes an enhanced multimodal biometric technique for a smart city that is based on score-level fusion. Specifically, the proposed approach provides a solution to the existing challenges by providing a multimodal fusion technique with an optimized fuzzy genetic algorithm providing enhanced performance. Experiments with different biometric environments reveal significant improvements over existing strategies. The result analysis shows that the proposed approach provides better performance in terms of the false acceptance rate, false rejection rate, equal error rate, precision, recall, and accuracy. The proposed scheme provides a higher accuracy rate of 99.88% and a lower equal error rate of 0.18%. The vital part of this approach is the inclusion of a fuzzy strategy with soft computing techniques known as an optimized fuzzy genetic algorithm.

## Introduction

The future of smart city security is based on multimodal biometrics. A biometric system is an automated system that recognizes a person related to behavioral or physiological characteristics, and it has made considerable progress in a variety of applications, such as surveillance, identification, access control, and protection. Facial characteristics, retinal characteristics, vein patterns, speech patterns, keystroke dynamics, nail bed, ear design, fingerprints, and other biological attributes have also been studied for verification purposes. Among these different traits, fingerprints seem to be a commonly used biometric trait. Additionally, the iris is the most accurate biometric because it is distinctive and consistent over time^1^.

While *unimodal* authentication technologies are more accurate, they only address a few issues, such as spoofing resistance and good privacy. Personal and biological problems, such as limited sample size and noise tracking devices, have a significant impact on the accuracy rate of unimodal biometric systems. Multimodal biometrics has been used to verify people to make authentication easier in smart city environments. Multimodal biometric systems have progressively been introduced to solve these issues. Supplementary traits derived from divergent modalities are used in multimodal biometric systems. Multimodal biometric identification systems outperform *unimodal* biometric security systems in terms of spoofing resistance and improving capabilities^2^.

The drawback of the existing approach is that the efficiency of most current multimodal biometric systems is hindered by contradictory classifier ratings. To address this problem, a new multi-biometric system based on a machine learning network is presented.

The most appropriate and effective technique for multimodal biometrics for smart cities has been stated, which presents a convergence of information at the scoring stage. The fusion of several biometric modalities focused on matching rankings is gaining popularity and appears to be a very promising approach to strengthen accuracy. Some score-level convergence techniques have been adopted thus far for this initiative^3^. Fuzzy techniques with optimization strategies offer enhanced security and accuracy of authentication systems^4^. The proposed multi-biometric authentication strategy uses fingerprint and iris biometric traits for recognition^5,6^. Initially, iris preprocessing is performed with iris localization, iris normalization, and feature extraction. The importance of the application of biometric recognition in smart environments is discussed in^7–9^.

Similarly, fingerprint preprocessing is performed with various strategies, such as image enhancement, ROI selection, and feature extraction. The fusion is performed at score level matching using an enhanced optimized fuzzy genetic algorithm (OFGA). This feature enables the simulation of the effects of specialized soft computing techniques such as a genetic collection of rules, adaptive fuzzy models, and other similar techniques used to combine similarity scores in a multimodal biometric system. Consequently, it has been demonstrated that combining rankings optimization algorithms and fuzzy systems outperforms other well-known strategies in multimodal biometric method verification or recognition^10^, irrespective of the nature of the biometric modality.

The major contribution of the proposed research is specified as follows:

- To develop an optimized fuzzy genetic algorithm for enhanced multimodal biometric fusion and develop an efficient multimodal biometric recognition system for smart cities
- To achieve better ranking optimization compared to other soft computing approaches
- To prove the efficiency of the proposed methodology, the proposed approach is compared against existing state-of-the-art approaches.

The remainder of the paper is organized as follows. Related works are provided in the second section. “Proposed methodology” section describes the proposed methodology that uses an optimized fuzzy genetic algorithm for score-level fusion to enhance recognition. “Results and discussion” section describes the result analysis with two standard datasets of fingerprints and irises. “Conclusion” section concludes the proposed research by highlighting the performance improvement.

## Related works

Experts in the field of multimodal biometric security have developed several methods. A brief overview of some significant contributions to the current literature is discussed in this section. Gavisidappa et al. ^11^. devised an efficient feature selection technique to decide the best-selected features for optimizing multimodal biometric identification efficiency. Following extracting features, the revised relief feature selection technique was used to deny irrelevant attributes and pick the best attributes. In an attempt to discover the closest miss and closest hit cases, a modified algorithm was used that used Chebyshev distance rather than just Manhattan distance.

Jagadiswary et al. ^12^. suggested a multimodal authentication system that uses feature extraction from various modalities, such as fingerprints, retinas, and finger veins. The RSA algorithm is used for key generation and encryption. The experimental analysis revealed that they have enhanced performance in terms of GAR of approximately 95.3% and FAR of approximately 0.01%. Vidya & Chandra^13^ present a novel security model for cloud-based encrypted storage that uses times of high-demand authentication by combining different biometric modalities from individuals and granting or denying access based on the results. ELBP is a modern texture-based dimensionality reduction technique that is proposed to represent entropy details in one dimension using a local binary pattern feature vector. There is no need for quantization with the ELBP function extraction method. Yang et al.^14^ examined the effect of feature composition on multi-biometric device matching results using a multi-biometric framework based on fingerprints and faces that uses feature-level fusion^15^. They use a percentage weight and a random projection-dependent transformation. It has also been demonstrated that allocating disproportionate amounts to features from various biometric features results in different matching outputs by changing this loss function.

Fuzzy logic has several common characteristics, such as allocated information representation and prototype approximation, as well as the capacity to interact with documents with ambiguity and inconsistency. Fuzzy logic tolerates data inaccuracy in the same way that neural networks tolerate noisy records. The mastering capability of a neural network is a fantastic way to change an expert's theory, and it automatically creates more fuzzy instructions and club capabilities to meet real-world requirements. This cuts down on both layout time and cost. The fuzzy logic framework, on the other hand, is likely to enhance the generalization features of a neural network by providing greater reliability^16^. When developing a multimodal biometric device, the fusion methodology adopted has a significant effect on its efficiency. Feature-level fusion is the most stable of the different fusion techniques. The feature set derived from multiple biometric features and used for multimodal device design could be inconsistent^17^.

Several methods using fuzzy basic logic to improve biometric fusion have been suggested in recent literature. In addition, fuzzy-based preference fusion is used on three biometrics where voting strategies are used. The fuzzy logic for fusion and the probability ratio-based complete fusion scheme were explored^18,19^.

For security purposes, Sujithra et al. ^20^. used multimodal biometrics such as heartbeat-based ECG signals as individual components in fingerprint systems. This has been used as a controlled instrument to capture the heartbeat pulse from twenty-two subjects' fingertips, as well as to assess the unimodal method of using the heartbeat alone. During the fusion phase of multimodal identification technologies, Selwal et al.^21^ developed a union operation of fuzzy correlations of modality templates. This method accomplishes both feature fusion and the translation of several templates into a unique, reliable, noninvertible template. The proposed methodology is irreversible, versatile, and has been tested on a bimodal biometric system that includes fingerprints and iris scans.

Tong et al.^22^ describe some intelligent algorithms with specific implementations to make cities smarter. The new urban framework for smart environments provides many opportunities to meet new challenges while solving concrete problems at the same time. Also, Huang et al.^23^ put the focus on multi-modal information perception using some machine learning algorithms. The experiment results show that the proposed methods and recognition approaches are effective and feasible. Pang et al.^24^ suggest a novel method based on the collaborative paradigm that allows multiple city digital twins (DT) using federated learning (FL). This approach allows sharing the local strategy and provides a global model in multiple iterations at different city DT systems. An interesting study based on an intelligent monitoring system is presented by Mabrouki et al.^25^. Experimental results carried out at various locations show some new interesting findings in the IoT-based motion intelligence monitoring system.

## Proposed methodology

Matching scores provide enough details to differentiate legitimate and impostor cases, and they are easily obtainable, so score-level fusion is widely used in multimodal biometric systems. Even without knowing the fundamental feature extraction and matching algorithms of each device, matching scores for a prespecified number of users can be produced provided several biometric systems.

**Figure 1:**
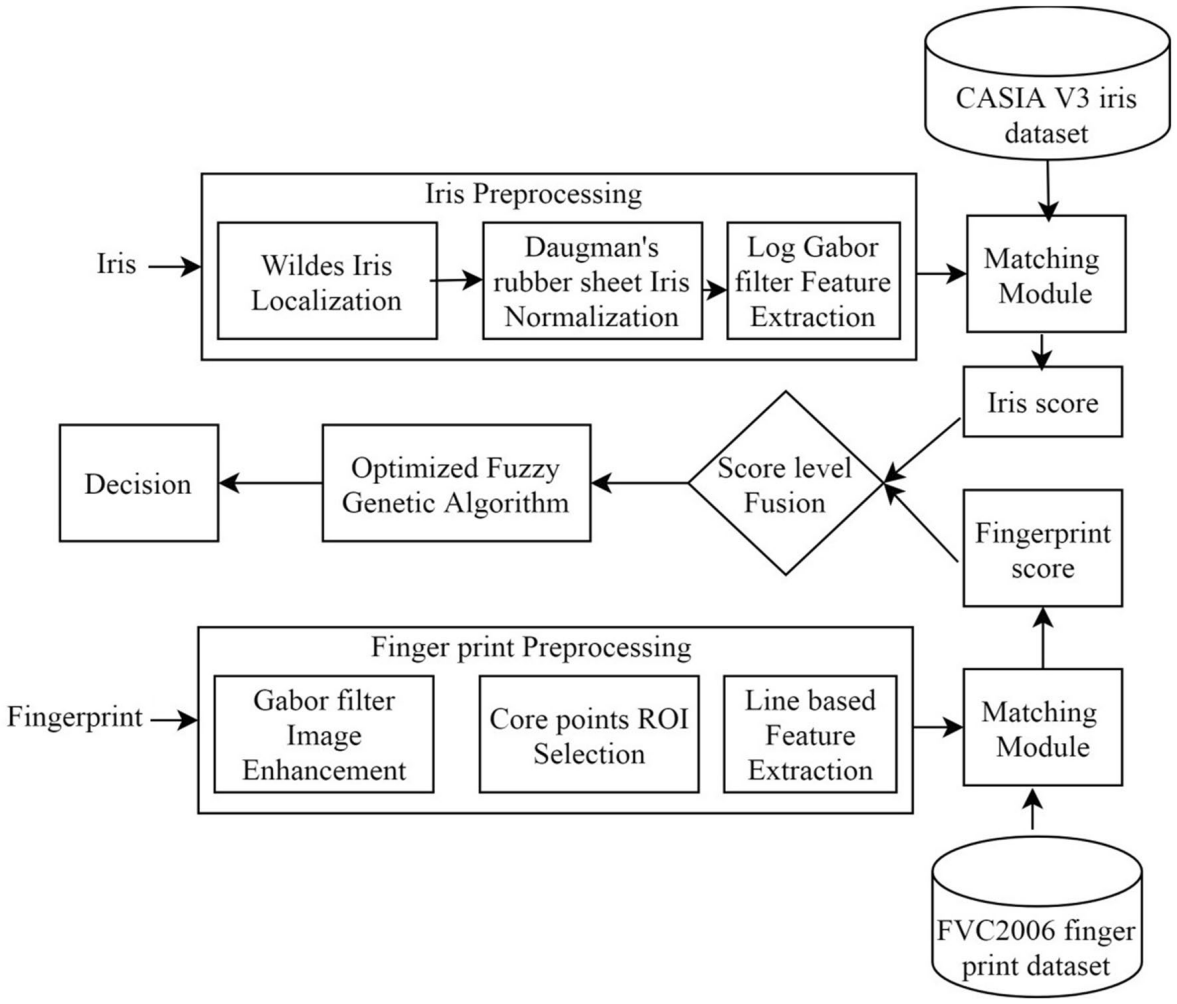
The flow of proposed approach for smart city.

The proposed methodology employs the score-level fusion technique based on an optimized fuzzy genetic algorithm. Initially, the finger and iris biometrics of the user are obtained and preprocessed. The feature extracted from the preprocessing step is matched against the biometrics stored in a database. The fusion of scores is done by using an optimized fuzzy genetic algorithm. The flow of the proposed approach is shown in Fig. 1.

### Iris preprocessing

The iris, which is part of ocular biometrics, is an externally noticeable yet secure organ with a special epigenetic pattern that remains stable during adulthood. As a result of this property, it is a critical methodology for use as a biometric for identification. If a person wants to be recognized by an iris recognition system in smart environments, their eyes are examined first, and then a prototype is produced. This template is then matched to other templates in a database before a match is found, or it remains unknown. The steps involved in iris preprocessing are iris localization, iris normalization, and feature extraction.

The proposed scheme uses the “*Wildes iris localization”* strategy. The characteristics of this approach are the combination of the Hough transform and edge map to localize iris and pupil boundaries. Initially, it will extract the edge map from the iris image and use the image intensity gradient as follows (1):

1 $$\left| {\Delta g(s,t)*i(x,y)} \right|$$

where $\Delta g(s,t)$ denotes the 2D Gaussian filter centered at *s,t* and $i(x,y)$ denotes the iris image with the locations *x* and *y*.

The subject was biased vertically for limbic boundary localization and horizontally for eyelid localization, suggesting that the subject's head was in a standing posture. Once the edge map is extracted, boundaries are extracted from pupil and limbic boundaries. For the iris image to be localized, with center (*x*, *y*) and radius r, the Hough transform function is defined as (2):

2 $$H(x,y,r) = \sum\limits_{i = 1}^{n} {(p,q,x,y,r)}$$

where (*p,q*) are pixel locations.

The normalization technique used in the proposed approach is Daugman’s rubber sheet model. The pixels from Cartesian to polar coordinates are designed as follows:

3 $$I(u(r,\theta ),v(r,\theta )) = I(r,\theta )$$

4 $$u(r,\theta ) = (1 - r)x_{p} (\theta )$$

5 $$v(r,\theta ) = (1 - r)y_{p} (\theta )$$

The iris feature extraction used in this proposed approach is the log Gabor filter technique^1^. The conventional choice of filters in feature extraction is normal Gabor filters. Typically, they suffer from two major drawbacks: their maximum bandwidth is limited to approximately one octave, and they are not ideal for obtaining broad spectrum content with maximal spatial localization. Thus, a logarithmic Gabor filter called the log-Gabor filter is introduced as an alternative to the normal Gabor filter. This log-Gabor filter is based on the fact that when viewed on a logarithmic frequency scale, the natural images can be better coded using filters having a Gaussian transfer function. When analyzed on a linear scale ranging, Gabor functions have a Gaussian kernel function. A log-Gabor filter's frequency response can be written as (6):

6 $$G(s) = \exp \left\{ { - 0.5 \times \frac{{\log \left( {\frac{s}{{s_{0} }}} \right)^{2} }}{{\log \left( {\frac{\sigma }{{s_{0} }}} \right)^{2} }}} \right\}$$

### Fingerprint preprocessing

Fingerprint preprocessing consists of three steps: image enhancement, region of interest (ROI) selection, and feature extraction.

A graphics file is used to read an image as input. To transform the image into a norm or assume a constant scale, bilinear interpolation is used. The region of interest is identified by the normalization technique. Initially, the input image is divided into various blocks $n \times n$, and the standard deviation can be obtained as $std(i)$. If the obtained standard deviation value is greater than the fixed threshold, the image can be marked as part of the fingerprint. The normalized image is given as

7 $$i(x,y) = i(x,y) - mean(i)$$

8 $$i(x,y) = i(x,y)/std(i)$$

9 $$i_{m} (x,y) = rm + i(x,y) \times sqrt(rv)$$

where *rm* denotes the required mean, *rv* denotes the required variance, and $sqrt$ denotes the square root function.

Gabor filter techniques are applied in addition to the above criteria to enhance the image. Normalization, segmentation of the ridge area, morphological elimination of small artifacts and approximation of the local orientation of ridges in a fingerprint are all part of the optimization phase. Thinning is applied to the binary image that has been collected.

ROI selection involves the identification of core points from the enhanced image. To obtain the region of interest, *n* pixels around the core point will be selected. Better to choose the value of *n* is 100 to 200 points so that adequate is around the core will be considered as ROI.

For perfect feature extraction from fingerprints, a line-based extraction algorithm^1^ is used. All associated line segments concerning all pixels are calculated using adaptive threshold analysis and the ridge tracing method. The extraction of minutiae is achieved by block extraction.

### Score-level fusion based on the optimized fuzzy genetic algorithm (OFGA)

The proposed technique uses OFGA to enhance the recognition of fingerprints and iris biometrics in the multimodal biometric recognition approach for smart cities. The higher morphological features of finger and iris biometrics are used for biometric evaluation. The matching rate used in this approach is defined as follows (10 and 11):

10 $$MR_{f} = w_{f} m_{f}$$

11 $$MR_{i} = w_{i} m_{i}$$

where $MR_{f}$ and $MR_{i}$ denote the matching rates of the fingerprint and iris, $w_{f}$ and $w_{i}$ denote the weights of the fingerprint and iris, where $m_{f}$ and $m_{i}$ denote the matching scores of the fingerprint and iris, respectively.

Among the various fusion regulations, the weighted sum rule is perceived as a variety of excellent ways of fusing the matching rates from various modalities to achieve better performance. The fusion rule is denoted here as a linear combination of the variable given in Eq. (12):

12 $$M_{S} = w_{i} m_{i} + w_{f} m_{f}$$

Optimized weight selection is necessary to implement better biometric fusion. Hence, the OFGA is proposed to achieve enhanced recognition. The OFGA method is a stochastic optimization method that is entirely based on a couple of parallels that seeks capability combined with genetic operations at some point during population evolution. The OFGA method is the combination of the fuzzy approach and a genetic algorithm where mutation and crossover are incorporated to minimize convergence. The goal of this proposed OFGA is a minimization of weights given in Eq. (13):

13 $$obj(Z) = \mathop {Min}\limits_{w} (w_{i} ,w_{f} )$$

where $obj(Z)$ is the objective function of feature Z with minimization of weight vector *w*.

The best way to achieve the overall performance is minimizing the equal error rate (EER). The steps involved in the genetic algorithm are:

- Initialization
- Fitness function
- Selection
- Crossover and mutation.

#### Initialization

The genetic algorithm performs randomization of the initial population as the very first step in the suggested optimization algorithm. Each function in the random sampling population is entirely vector-based and reflects all methods. The duration of the population is kept constant in this method.

#### Fitness function

In this proposed approach, EER and accuracy are considered to be the fitness function. A higher value of accuracy and a lower EER denote enhanced biometric recognition. Each member of the population is assigned a fitness value that is more closely related to the fitness function. As a result, the fitness value reflects the importance of each function and contributes to the convergence of the global value at the end of the mission.

#### Fuzzy clustering approach

Fuzzy clustering is a type of clustering where each data point can be allocated to multiple clusters. The population selection is denoted by an OFGA algorithm, where for every population $y = \left( {y_{1} ,y_{2} ,y_{3} ,,y_{n} } \right)$, it defines the rules to separate the data into various clusters $C = \left( {c_{1} ,c_{2} ,c_{3} ,,c_{m} } \right)$ to minimize the data feature $O_{x}$ where the fuzziness is limited by the factor $x$. The partition matrix is denoted by $W = w_{ij}$, which indicates that element $y_{i}$ belongs to $C_{j}$ (14):

14 $$\mathop {\arg \_\min }\limits_{C} = \sum\limits_{i = 1}^{i = n} {\sum\limits_{j = 1}^{j = m} {w_{ij} \left| {y_{i} - c_{j} } \right|} }^{2}$$

where

15 $$w_{ij} = \frac{1}{{\sum\limits_{k = 1}^{m} {\left( {\frac{{y_{i} - c_{j} }}{{y_{i} - c_{k} }}} \right)^{{\frac{2}{m - 1}}} } }}$$

For the next decade, the entities cluster with the highest fitness values is considered. The cluster with the lowest average has to be the worst, and it is excluded during the next generation. The features of this magnificence are advantageous for fast searches and extending the discovery process within the search room. This agency could serve as a transition zone for some exploitation and exploration. Although significant changes are made to them to search for better alternatives, the function with a low objective function is not removed.

*Crossover and mutation* are very important parameters in chromosome diversification. Then,$p_{c}$ and $p_{m}$ denote the probability of crossover and mutation, respectively. To acquire the strengthened solution, the importance of $p_{c}$ and $p_{m}$ in phase could dramatically change the convergence growth rate. The crossover and mutation operators' corresponding correlations and probabilities are applied to the global solution. The solution is specified in Eq. (16):

16 $$p_{i}^{j} = y_{i}^{j - 1} \times rand\left( {y_{i}^{j - 1} - x_{i}^{j - 1} } \right)$$

where $p_{i}^{j}$ represents the probability of crossover with i = c and the probability of mutation with *i* = *m*; $\left( {x_{i}^{j} ,y_{i}^{j} } \right)$ denotes the degree to which random selections of $p_{c}$ and $p_{m}$ are made. The intervals of crossover and mutation are $[0,1][0,1]$ and $[0,0.1]$, respectively.

## Results and discussion

When compared to unimodal biometric authentication, multimodal biometric authentication proved to have enhanced security and privacy. Fingerprint and iris recognition are used in the multimodal biometric system for smart city environments.

### Experimental setup

The proposed work is simulated using a Python programming language with 6 GB RAM and an*i5 processor*. The data used in the proposed approach are the CASIA iris V3 dataset and the FVC2006 fingerprint dataset. CASIA-IrisV3 is divided into three subgroups: CASIA-Iris-Interval, CASIA-Iris-Lamp, and CASIA-Iris-Twins. CASIA-IrisV3 comprises 22,034 image features from over 700 different subjects. Iris images were captured using infrared light illumination and are 8 bit gray-level JPEG files. The FVC2006 competition aims at evaluating fingerprint authentication tools. Authorized participants were given a subset of fingerprint observations collected with different sensors to fine-tune the specifications of their algorithms. The benchmark will consist of four distinct databases given by the organizers: DB1, DB2, DB3, and DB4. That database spans 150 fingers and includes 12 specimens per finger. The notations used in the proposed approach are false positives (FPs), true negatives (TNs), false negatives (FNs), and true positives (TPs)^26^.

### Performance evaluation on CASIA V3 and FVC2006 datasets

The effectiveness of the proposed approach is based on the following different metrics.

- *False Acceptance Rate (FAR):* This refers to the likelihood of a system incorrectly accepting a nonregistered or unauthorized user.
  17 $$FAR = \frac{FP}{{TN + FP}}$$
- *False Rejection Rate (FRR):* This refers to the likelihood of a system incorrectly rejecting a nonregistered or unauthorized user.
  18 $$FRR = \frac{FN}{{TP + FN}}$$
- *True positive rate (TRR):* This indicates the likelihood of a device authorizing the registered user. It is also known as sensitivity.
  19 $$TPR = \frac{TP}{{TP + FN}}$$
- *True Negative Rate (TNR):* This determines the likelihood of the authorized user being approved by a system. It is otherwise defined as recall or specificity.
  20 $$TNR = \frac{TN}{{TN + FP}}$$
- *Accuracy:* Registered users permitted at a rate proportional to the number of attempts they made.
  21 $$Accuracy = \frac{{\left( {TP + TN} \right)}}{{\left( {TP + TN + FP + FN} \right)}}$$
- *Equal error rate (EER):* The rate at which FAR is equal to FRR is known as EER.
- *Precision:* This indicates the ratio of positive instances found among all positives mentioned. It is otherwise denoted as a positive predictive value.

The efficiency of the proposed approach is analyzed for the iris and fingerprint at various samples. Table 1 and Table 2 specify the analysis on various parameters with different data densities without OFGA and with OFGA (N is number of iris and fingerprint samples).

**Table 1:** Performance evaluation on CASIA V3 and FVC2006 datasets without OFGA.

N	TP	FN	FP	TN	FAR (%)	FRR (%)	TPR (%)	TNR (%)	Precision	Accuracy (%)
100	100	0	0	100	0	0	100	100	100	100
300	300	0	0	300	0	0	100	100	100	100
500	499	1	1	499	0.2	0.2	99.8	99.8	99.8	99.8
800	797	3	2	798	0.25	0.38	99.63	99.75	99.75	99.63
1000	997	3	3	997	0.3	0.3	99.7	99.7	99.7	99.7
1300	1296	4	4	1296	0.31	0.31	99.69	99.69	99.69	99.69
1500	1495	5	5	1495	0.33	0.33	99.67	99.67	99.67	99.67
1800	1792	8	8	1792	0.44	0.44	99.56	99.56	99.56	99.56
2100	2089	11	12	2088	0.57	0.52	99.48	99.43	99.43	99.48

**Table 2:** Performance evaluation on CASIA V3 and FVC2006 datasets with OFGA.

N	TP	FN	FP	TN	FAR (%)	FRR (%)	TPR (%)	TNR (%)	Precision	Accuracy (%)
100	100	0	0	100	0	0	100	100	100	100
300	300	0	0	300	0	0	100	100	100	100
500	500	0	0	500	0	0	100	100	100	100
800	798	2	1	799	0.13	0.25	99.75	99.88	99.87	99.81
1000	998	2	1	999	0.18	0.18	99.8	99.9	99.9	99.85
1300	1296	4	2	1298	0.15	0.31	99.69	99.85	99.85	99.77
1500	1497	3	1	1499	0.07	0.2	99.8	99.93	99.93	99.87
1800	1792	7	5	1795	0.28	0.39	99.61	99.72	99.72	99.67
2100	2090	10	8	2092	0.38	0.48	99.52	99.62	99.62	99.57

Similarly, the biometric system with lower equal error rate provides better performance. The EER is most important metric in biometric recognition system for measuring enhanced performance. The EER of the proposed Iris and fingerprint recognition without OFGA is 0.33%. Similarly, the EER of the proposed Iris and Fingerprint recognition with OFGA is 0.18%.

The accuracy of the proposed approach is 99.73% without an optimization algorithm and when the optimization process included the accuracy is enhanced to 99.88%. The average performance analysis of the proposed approach in terms of FAR, FRR, TPR, TNR, accuracy, EER, and precision is specified in Table 3.

**Table 3:** Average performance evaluation.

Modalities	FAR (%)	FRR (%)	TPR (%)	TNR (%)	Accuracy (%)	EER (%)	Precision
Iris and Finger print without OFGA	0.26	0.27	99.73	99.74	99.74	0.33	99.73
Iris and Finger print with OFGA	0.13	0.20	99.79	99.88	99.83	0.18	99.88

**Figure 2:**
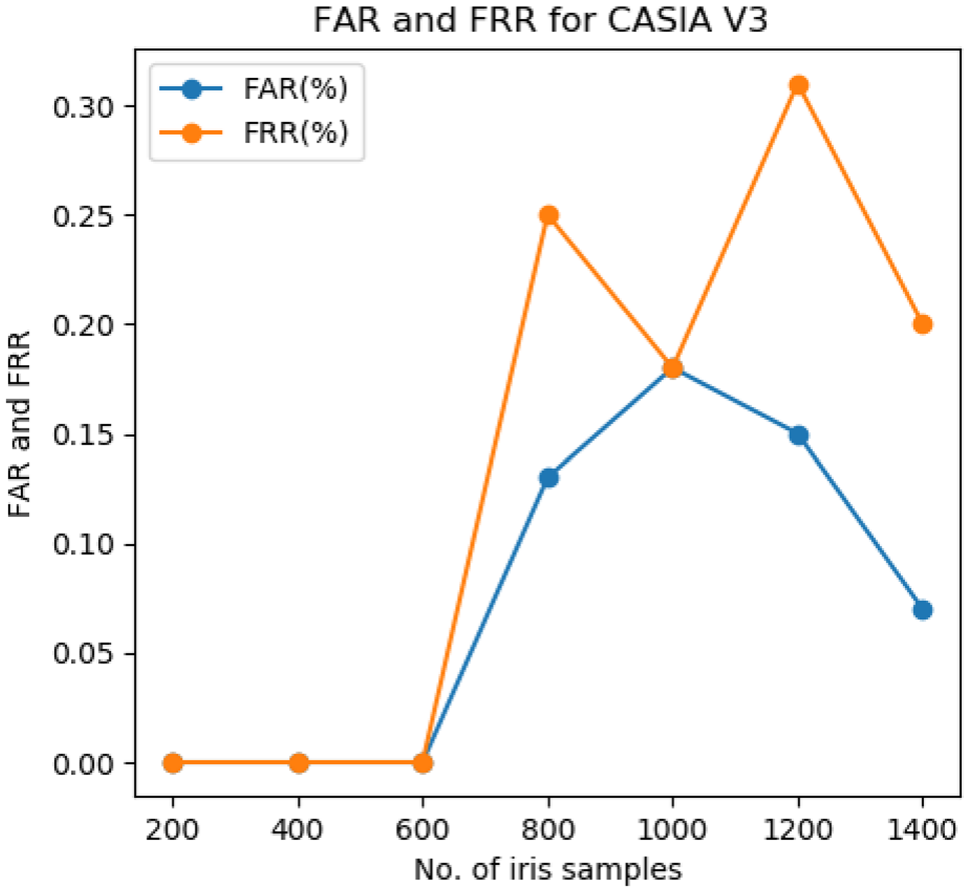
FAR and FRR of CASIA V3 iris dataset.

The FAR and FRR of the CASIA V3 iris dataset are shown in Fig. 2. The curve shows that the average FAR and FRR rate of the iris dataset are 0.13% and 0.20%, respectively. The TPR and TNR is an important metric for a biometric recognition system. The TPR and TNR of the proposed approach without OFGA is 99.79% and 99.88% respectively. Similarly, the TPR and TNR of the proposed approach with OFGA are 99.79% and 99.88% respectively.

**Figure 3:**
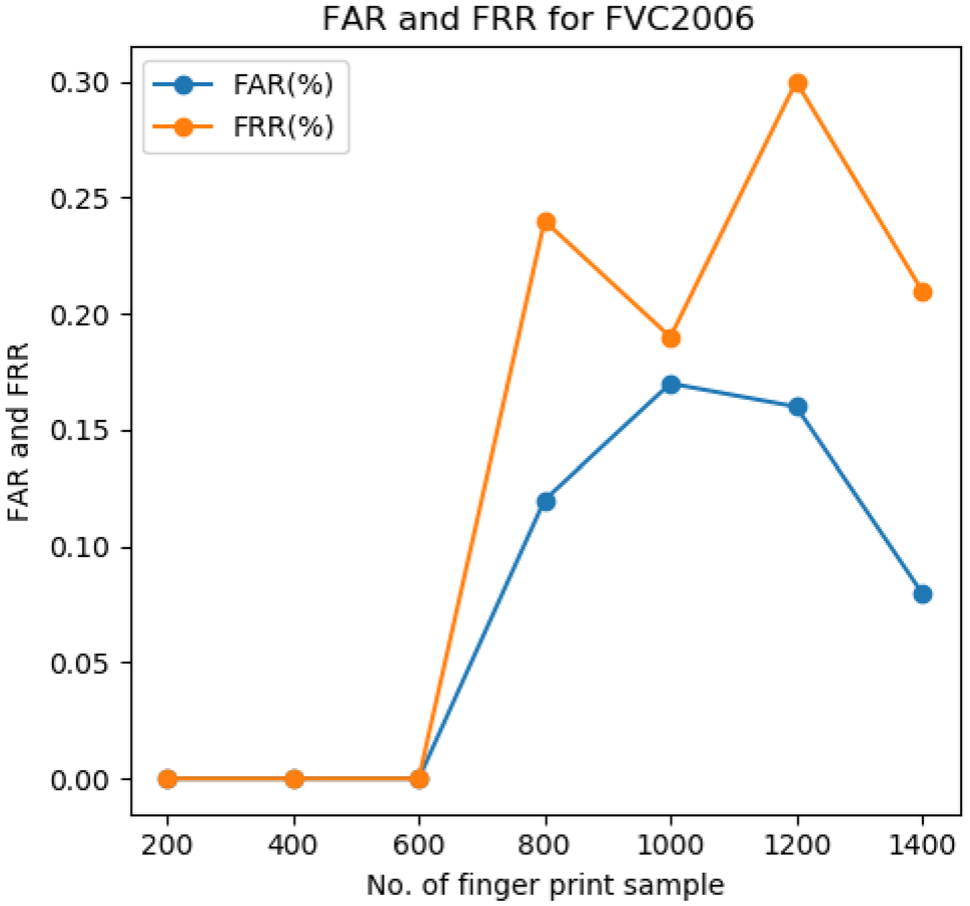
FAR and FRR of FVC2006 fingerprint dataset.

Similarly, the FAR and FRR of the FVC2006 fingerprint dataset are 0.14% and 0.20%, respectively (Fig. 3). The point where the FAR equals FRR is known as EER. Systems with a lower EER rate have higher reliability and security The FAR and FRR of the iris dataset shows the first line of equals at 0.18 which is lower compared to that of existing approaches.

**Figure 4:**
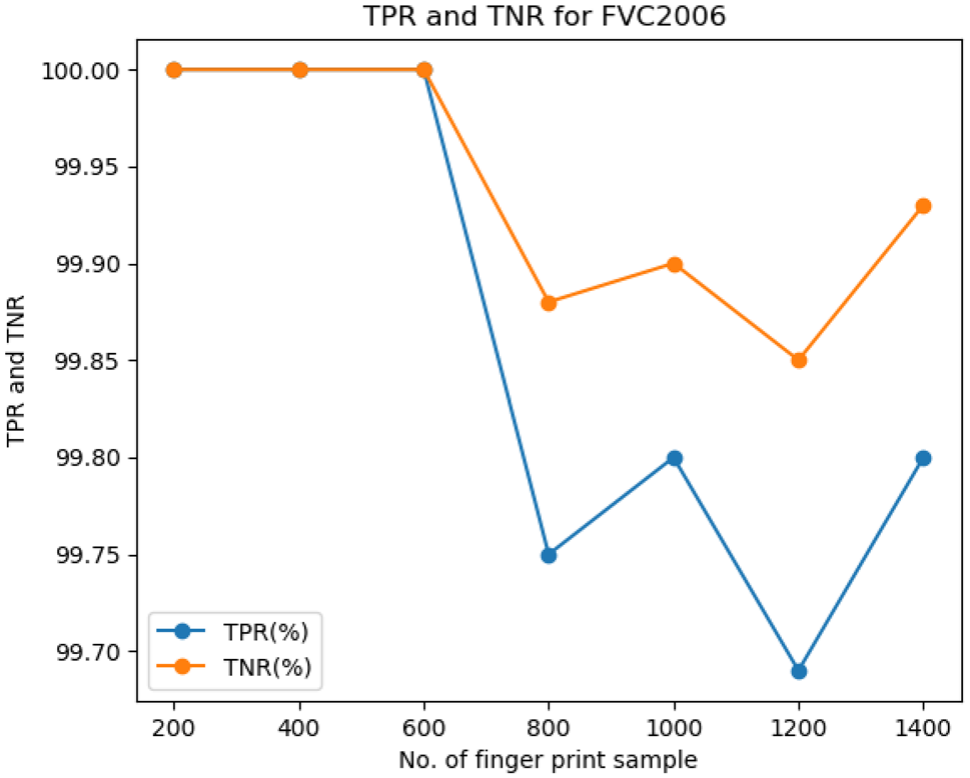
TPR and TNR of FVC2006 fingerprint dataset.

**Figure 5:**
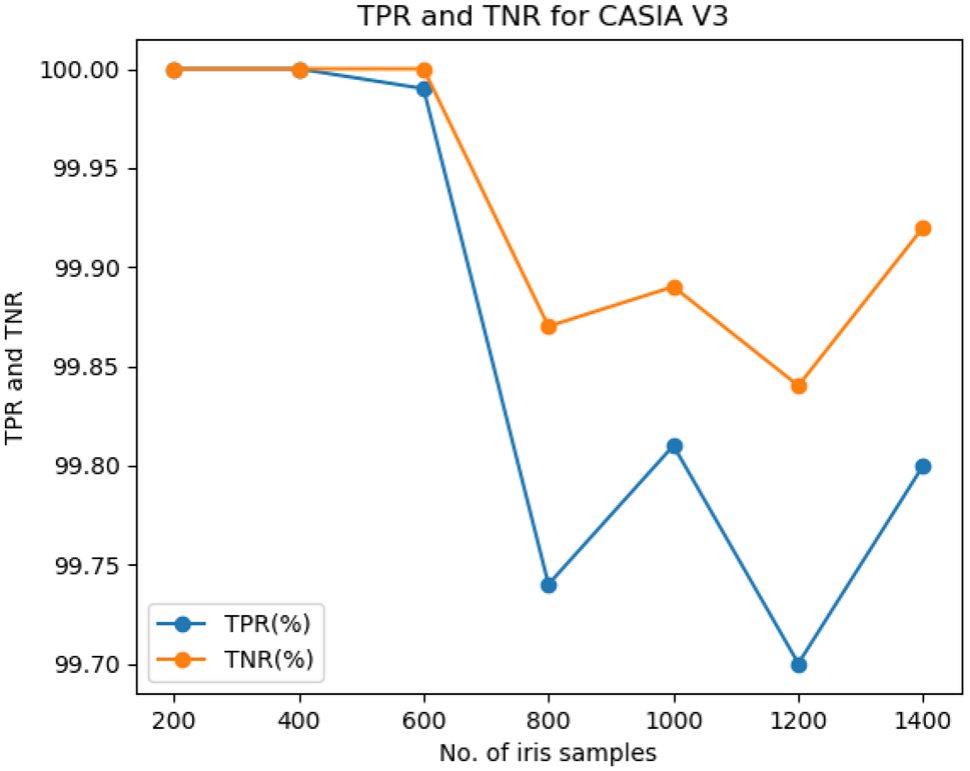
TPR and TNR of CASIA V3 iris dataset.

The comparison of the proposed approach against the TPR and TNR of the fingerprint dataset and iris dataset is shown in Figs. 4 and 5 respectively.

### Comparative analysis

The six methods considered for comparison are Gavisiddappa et al.^11^, where the author uses fingerprints and irises with a modified relief approach that achieves an accuracy of approximately 97.09%. The second method for comparison is the Jagadiswary et al.^12^ approach of the fusion level multimodal combination approach, which achieves a higher accuracy of 87.6%. Vidya & Chandrause^13^ ELBP method feature extraction achieved higher accuracy in multimodal biometrics of 91.0%. The next method of comparison is Yang et al.^14^ feature-level matching, given a higher accuracy of 90%. Similarly, Malarvizhi et al.^10^ proposed a multimodal strategy based on soft computing approaches called an adaptive fuzzy genetic algorithm. The accuracy of their approach is 96%. Selwal et al.^21^ proposed a biometric recognition model based on a union operation fuzzy relation strategy. The comparison of the proposed approach with various state-of-the-art existing approaches in terms of accuracy and EER are shown in Table 4. The proposed approach is compared for FAR, FRR, Accuracy, and EER with the existing approaches.

**Table 4:** Comparison of proposed approach with existing approaches.

Approaches	FAR (%)	FRR (%)	Accuracy (%)	EER (%)
Gavisiddappa et al.^11^	9.87	11.89	97	0.23
Jagadiswary et al.^12^	0.01	0.27	87	0.37
Vidya & Chandrause^13^	0.32	0.33	91	0.32
Yang et al.^14^	0.38	0.27	90	0.33
Malarvizhi et al.^10^	0.58	0.02	96	0.22
Selwal et al.^21^	3.30	3.39	97	0.20
Proposed approach	0.13	0.20	99.83	0.18

The multimodal biometric system with higher accuracy seems to be more applicable for real-time applications such as banking, passport, financial, login applications, etc. Feature selection is an important aspect of the multimodal biometric security method in this study. Each biometric image contains a large number of features and a large amount of data, resulting in the “curse of dimensionality” issue. As a result, feature selection is critical to optimize the features that are most suitable for classification. The accuracy of the system obtained based on their model is 97.3%. The average accuracy of the proposed OFGA based multimodal biometric system is 99.88%.

**Figure 6:**
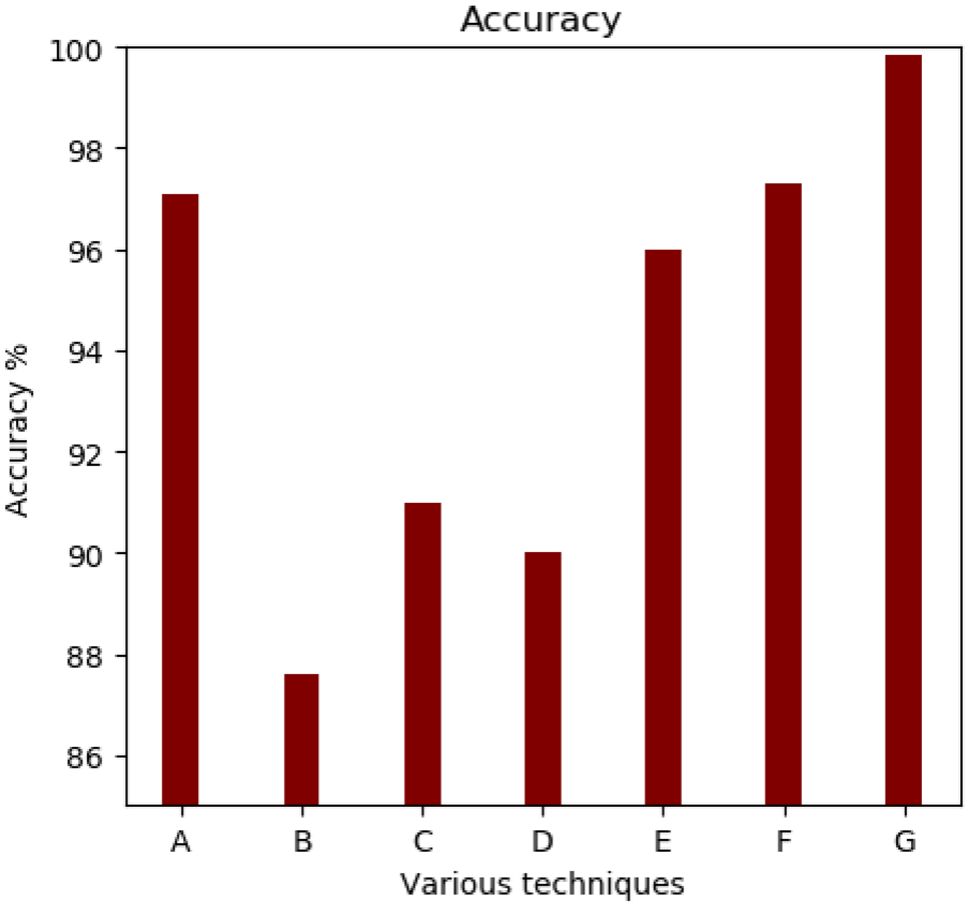
Comparative analysis-Accuracy comparison.

**Figure 7:**
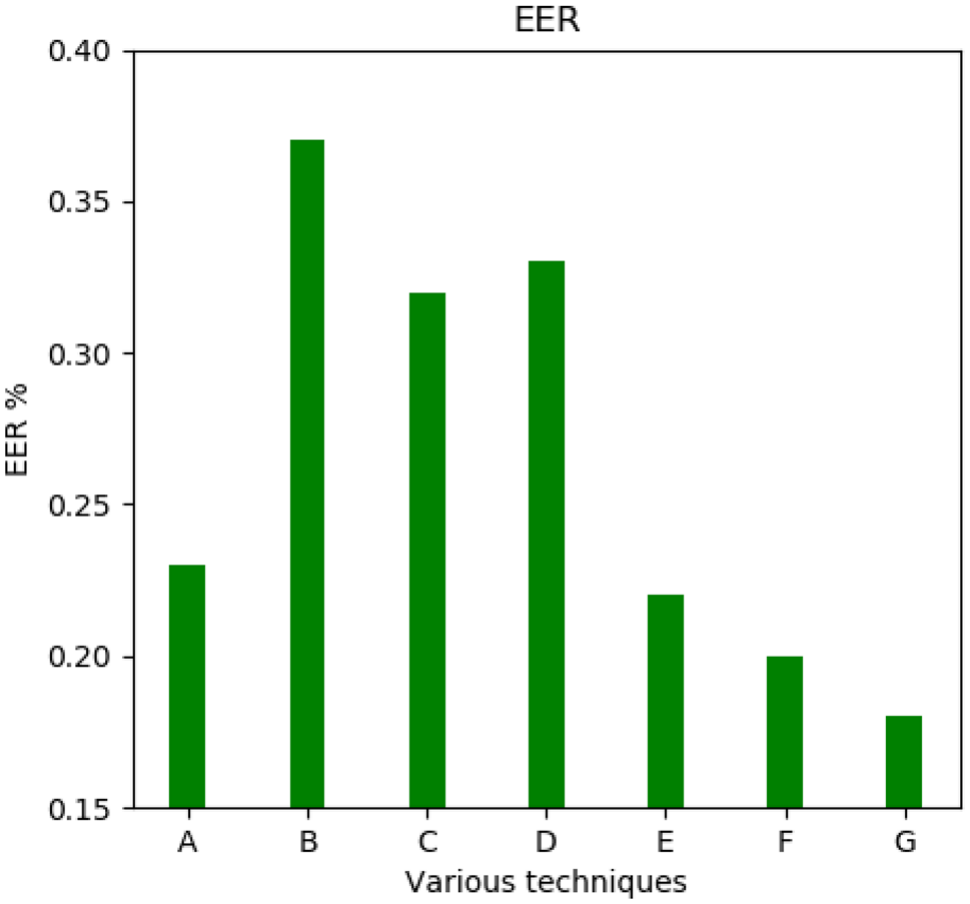
Comparative analysis-EER comparison.

The accuracy comparison is depicted in Fig. 6 (*A* = *ref*^11^*; B* = *ref*^12^*; C* = *ref*^13^*; D* = *ref*^14^*; E* = *ref*^10^*; F* = *ref*^21^*; G* = *Proposed OFGA method*). Similarly, the proposed approach is compared for EER with the state-of-the-art approaches. The average EER of the proposed method is 0.18%. The ERR comparison is depicted in Fig. 7.

## Conclusion

The proposed approach for smart cities uses novel multimodal biometrics that uses fingerprint and iris biometric traits. The prime objective of this method is to incorporate enhanced fusion techniques with fuzzy models and soft computing approaches. Multimodal biometrics uses an optimized fuzzy genetic algorithm for an effective fusion strategy and accurate biometric recognition. Initially, preprocessing is performed for both fingerprint and iris modalities.

The effective features extracted from the feature extraction mechanism are given as input in the score-level fusion step. The optimized fuzzy genetic algorithm provides enhanced performance in terms of higher accuracy, higher true positive rate, higher true negative rate, lower false acceptance rate, lower false rejection rate, and lower equal error rate. The accuracy of the proposed approach is compared with four state-of-art existing approaches.

The comparison showed that the proposed approach provides an enhanced accuracy of approximately 99.89% and a lower equal error rate of approximately 0.18%. The matching score obtained for fingerprint and iris biometrics in a large number of subjects indicates that this integration has real potential.

This method is advantageous for improving security because it improves overall efficiency and biometrics' underlying aliveness value in smart cities. In the future, more biometric modalities will be included in addition to fingerprints and irises to improve smart environments. Additionally, an enhanced classification algorithm will be used to boost accuracy.

## Data Availability

As human data is used in the study, although it is a publically available dataset, all methods were carried out in accordance with relevant guidelines and regulations. All the fingerprints used in this work are taken from the dataset which is publically available. Source of dataset CASIA IRIS V3: http://biometrics.idealtest.org/findTotalDbByMode.do?mode=Iris#/datasetDetail/3. Source of dataset FVC2006 DATASET: http://bias.csr.unibo.it/fvc2006/download.asp.

## References

[1] Rajasekar V, Premalatha J, Sathya K, Saračević M (2021). Secure remote user authentication scheme on health care, IoT and cloud applications: a multilayer systematic survey. Acta Polytechnica Hungarica.

[2] Wencheng Y, Wang S, Hu J, Zheng G, Valli C (2019). Security and accuracy of fingerprint-based biometrics: a review. Symmetry.

[3] Gavrilova, M. L., Ahmed, F., Azam, S., Paul, P. P., Rahman, W., Sultana, M., & Zohra F.T. Emerging trends in security system design using the concept of social behavioral biometrics. In *Information Fusion for Cyber-Security Analytics* 229–251 (Springer, 2017).

[4] Donghoon C, Garg S, Hasan M, Mishra S (2020). Cancelable multibiometric approach using fuzzy extractor and novel bitwise encryption. IEEE Trans. Inf. Forensics Secur..

[5] Rajasekar V, Premalatha J, Sathya K (2020). Multi factor signcryption scheme for secure authentication using hyper elliptic curve cryptography and biohash function. Bull. Polish Acad. Sci. Tech. Sci..

[6] Galterio MG, Angelic SS, Hayajneh T (2018). A review of facial biometrics security for smart devices. Computers.

[7] Menon V, Jayaraman B, Govindaraju V (2013). Enhancing biometric recognition with spatiotemporal reasoning in smart environments. Pers. Ubiquit. Comput..

[8] Ryo, O., & Yasushi, Y. Smart Device-based Multimodal Biometric Authentication with the Function for Environment Recognition. In* Proceedings of international symposium on computing and networking (CANDAR)*, IEEE Book Series: International Symposium on Computing and Networking 495–498 (2015).

[9] De Marsico M, Mecca A, Barra S (2019). Walking in a smart city: investigating the gait stabilization effect for biometric recognition via wearable sensors. Comput. Electr. Eng..

[10] Malarvizhi N, Selvarani P, Raj P (2020). Adaptive fuzzy genetic algorithm for multi biometric authentication. Multimed. Tools Appl..

[11] Gavisiddappa G, Mahadevappa S, Mohan PC (2020). Multimodal biometric authentication system using modified ReliefF feature selection and multi support vector machine. Int. J. Intell. Eng. Syst..

[12] Jagadiswary D, Saraswady D (2016). Biometric authentication using fused multimodal biometric. Procedia Comput. Sci..

[13] Vidya SB, Chandra E (2019). Entropy based Local Binary Pattern (ELBP) feature extraction technique of multimodal biometrics as defense mechanism for cloud storage. Alex. Eng. J..

[14] Yang W, Wang S, Zheng G, Valli C (2019). Impact of feature proportion on matching performance of multibiometric systems. ICT Express.

[15] Rajasekar V, Premalatha J, Sathya K (2021). Cancelable Iris template for secure authentication based on random projection and double random phase encoding. Peer-to-Peer Netw. Appl..

[16] Al-Hmouz R, Pedrycz W, Daqrouq K, Morfeq A (2018). Development of multimodal biometric systems with three-way and fuzzy set-based decision mechanisms. Int. J. Fuzzy Syst..

[17] Manvjeet, K., & Sofat, S. Fuzzy vault template protection for multimodal biometric system. In *2017 International Conference on Computing, Communication and Automation (ICCCA IEEE)*, Greater Noida, India 1131–1135 (2017).

[18] EmadMajeed H, Abbood N, Alani AA (2019). Fuzzy logic decision fusion in a fingerprints based multimodal biometric system. J. Eng. Appl. Sci..

[19] Taranpreet, K., & Kaur, M. Cryptographic key generation from multimodal template using fuzzy extractor. In *2017 Tenth International Conference on Contemporary Computing (IC3 IEEE)*, Greater Noida, India, Aug 10–12 (2017).

[20] Sujitha V, Chitra D (2019). A novel technique for multi biometric cryptosystem using fuzzy vault. J. Med. Syst..

[21] Selwal A, Gupta SK, Kumar S (2016). A scheme for template security at feature fusion level in multimodal biometric system. Adv. Sci. Technol. Res. J..

[22] Tong Z, Ye F, Yan M, Liu H, Basodi S (2021). A survey on algorithms for intelligent computing and smart city applications. Big Data Min. Anal..

[23] Huang H, Lin J, Wu L, Fang B, Wen Z, Sun F (2020). Machine learning-based multi-modal information perception for soft robotic hands. Tsinghua Sci. Technol..

[24] Pang J, Huang Y, Xie Z, Li J, Cai Z (2021). Collaborative city digital twin for the COVID-19 pandemic: a federated learning solution. Tsinghua Sci. Technol..

[25] Mabrouki J, Azrour M, Fattah G, Dhiba D, Hajjaji SE (2021). Intelligent monitoring system for biogas detection based on the Internet of Things: Mohammedia, Morocco city landfill case. Big Data Min. Anal..

[26] Rajasekar, V., Premalatha, J., & Sathya, K. Enhanced biometric recognition for secure authentication using Iris preprocessing and hyperelliptic curve cryptography. *Wirel. Commun. Mobile Comput*. Art. No. 8841021 (2020).

